# Porous Microparticles Containing Raloxifene Hydrochloride Tailored by Spray Freeze Drying for Solubility Enhancement

**DOI:** 10.15171/apb.2018.026

**Published:** 2018-06-19

**Authors:** Seyyed Pouya Hadipour Moghaddam, Sajjad Farhat, Alireza Vatanara

**Affiliations:** ^1^Department of Pharmaceutics and Pharmaceutical Chemistry, School of Pharmacy, University of Utah, Salt Lake City, UT 84112, USA.; ^2^Utah Center for Nanomedicine, Nano Institute of Utah, University of Utah, Salt Lake City, UT 84112, USA.; ^3^Department of Pharmaceutics, School of Pharmacy, Tehran University of Medical Sciences, Tehran, Iran.

**Keywords:** Dissolution profile, Porous microparticles, Raloxifene, Solubility, Spray freeze drying

## Abstract

***Purpose:*** The goal of this study was to improve the solubility and dissolution behavior of Raloxifene Hydrochloride (RH) using Spray Freeze Drying (SFD) technique.

***Methods:*** For achieving this goal, series of samples containing RH with polyvinylpyrrolidone (PVP) or hydroxypropyl beta cyclodextrin (HPβCD) used as solubility enhancers were prepared and microparticles were formed via SFD. The resultant microparticles were physicochemically characterized. Morphology of the microparticles were observed using Scanning Electron Microscopy (SEM). High Performance Liquid Chromatography (HPLC) was used for analyzing the solubility and dissolution profile of the samples.

***Results:*** Fourier Transmission Infrared (FTIR) spectra showed that SFD processed compositions did not affect chemical structure of RH. SEM and Thermal Gravimetric Analysis (TGA) revealed that the fabricated spherical and highly porous microparticles were in amorphous state. SFD processed powders showed superior solubility and dissolution behavior; where, 80% of the drug was dissolved within 5 minutes.

***Conclusion:*** SFD method can be a promising alternative for enhancing the solubility of poorly water soluble compounds.

## Introduction

Raloxifene Hydrochloride (RH) is a Selective Estrogen Receptor Modulator (SERM) acts as an agonist on bone and liver. However, Raloxifene shows antagonistic effects on breast and uterus. Hence, Raloxifene has been approved for the prevention of osteoporosis as well as reducing the risk of invasive breast cancer in post-menopausal women.^1^ RH shows very low oral bioavailability (*c.a.* 2% of the given dose) which is mostly due to its extensive first pass hepatic metabolism *via* glucuronide conjugation as well as incomplete dissolution as the drug is poorly water-soluble.^2,3^ This drug is categorized as class II of Biopharmaceutical Classification System (BCS).^4^ Accordingly, its low bioavailability seems to be enhanced by increasing its solubility.^5,6^

Several techniques can be used to improve solubility of drugs such as co-grinding, salt formation, spray drying, and supercritical fluid processing.^7-10^ In order to increase the solubility of RH, different methods have been investigated to date. Co-grinding of RH with different superdisintegrants such as polyvinylpyrrolidone (PVP), hydroxypropylmethyl cellulose, crospovidone, croscarmellose sodium, and sodium starch glycolate have been reported and resulted in considerable improvement of drug solubility in most cases.^7,11^ Spray freeze drying (SFD) is relatively a novel technique for pharmaceutical particle engineering and drying of foods as well as bioproducts.^12,13^ In this process, a feed solution is atomized by a nozzle over/in a cryogenic medium and frozen droplets are lyophilized in low pressure and temperature. In comparison to other particle engineering processes, SFD grants better control on different aspects of particle properties. This method can produce highly porous and low density particles. It has been reported that SFD could create particles forty times higher in specific surface area and one ninth lower in density versus spray drying technique.^14^ Consequently, SFD is now an authentic approach for enhancement of particle properties like surface area and increasing dissolution of different drugs.

Many factors can influence particle characteristics produced by this method such as composition, total solid content of liquid feed solution, spraying rate of liquid feed, distance between nozzle, and cryogenic liquid surface.^15,16^

In the present study, the effect of SFD method on dissolution rate of RH was studied in the presence of PVP and HPβCD as solubility enhancers. PVP has been widely used for solubility improvement of water-insoluble drugs such as piroxicam, furosemide, praziquantel, and celecoxib.^17-20^ Cyclodextrin (CD) derivatives are cyclic oligosaccharides comprise hydrophilic outer part and hydrophobic internal cavity which can form a complex with various drugs. CDs have been used to increase water solubility and dissolution rate of low water soluble drugs such as miconazole, doxorubicin, and naproxen.^21-23^ Complexation of RH with Hydoxybutenyl beta Cyclodextrin (HBenβCD) has been shown to be effective in increasing solubility and dissolution rate of the drug.^24,25^

## Materials and Methods

RH was received from Glochem (India). PVP K30 and HPβCD were purchased from Sigma Aldrich (Germany). Ethanol and acetonitrile of HPLC grade were obtained from Merck (Germany) and the liquid nitrogen were received from Sabalan (Iran).

### Formation of RH Microparticles via SFD Method

**Figure 1:**
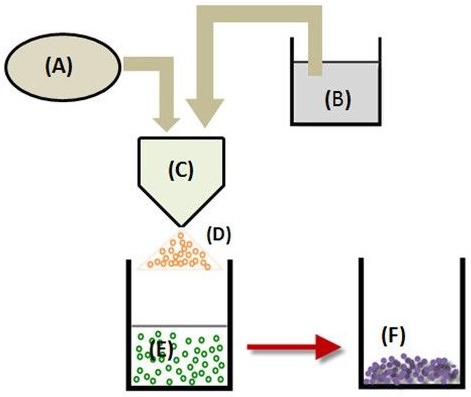
Schematic diagram of spray freeze drying apparatus. (A) Compressed air inlet, (B) Solution cell, (C) Two fluid nozzle, (D) Drug containing droplets, (E) Frozen particles in liquid nitrogen, and (E) Powder collected after freeze drying.

Hydroethanolic (4:1) solvent was selected to dissolve the drug and excipients. Samples were prepared in compositions according to the Table 1. To produce spray freeze dried powders, the feed solution was loaded into the solution cell and sprayed 10 cm above the surface of 300 mL liquid nitrogen through a two-fluid nozzle with a flow rate of 6 and 12 mL/min at the pressure of 6 bars provided by an air pump. Figure 1 indicates a schematic diagram of the spraying set up used in this study. The resultant suspension (frozen droplets of the solution in liquid nitrogen) was transferred into the freeze dryer (Christ, The Netherlands). Vacuum was applied as soon as all nitrogen was evaporated. During the first 24 h, the pressure was set at 0.005 mbar and the shelf temperature was fixed at -70 °C. During the second 24 h, the shelf temperature was gradually raised to 20 °C. After removing the samples from the freeze drier, they were stored over silica gel in a desiccator at room temperature.

**Table 1:** Composition of spray freeze dried formulations

**Run No.**	**RH (mg)**	**HPβCD (mg)**	**PVP (mg)**	**Flow Rate (mL/min)**
**F**_1_	112.50	225	-	6
**F**_2_	75			6
**F**_3_	56.25			6
**F**_4_	112.50			12
**F**_5_	75			12
**F**_6_	56.25			12
**F**_7_	112.50	-	225	6
**F**_8_	75			6
**F**_9_	56.25			6
**F**_10_	112.50			12
**F**_11_	75			12
**F**_12_	56.25			12

RH: Raloxifene Hydrochloride HPβCD: Hydroxypropyl Beta Cyclodextrin PVP: Polyvinylpyrrolidone

### Thermal Analysis

Thermal analysis of RH, HPβCD, PVP, and selected SFD processed samples were performed using a PL-DSC apparatus (polymer laboratories, UK). Approximately, 5 mg of the samples were sealed firmly and scanned under dry nitrogen atmosphere at the heat rate of 10 °C per minute from 10 to 350 °C.

### Scanning Electron Microscopy (SEM)

A Philips Model XL30 scanning electron microscope (Philips, The Netherlands) was used to obtain the SEM images. The samples were glued onto aluminum stages using double adhesive carbon conducting tape and coated with gold–palladium at room temperature before the examination. The accelerator voltage for scanning was 25.0 kV.

### Fourier Transform Infrared Spectroscopy (FTIR)

FTIR spectra were recorded with a spectrophotometer (Mega-IR, 550, Nicolet, USA) in the range of 400-4000 cm^-1^, using a resolution of 4.000 cm^-1^ and 4 scans. Samples were diluted with KBr at concentration of 1% and pressed to obtain self-supporting disks.

### Solubility and Dissolution Studies

An excess amount of RH formulations was added to 20 mL of freshly prepared deionized water and rotated at 100 rpm in a water bath (Dorsa, Iran) for 150 min. Then, the samples were filtered and analyzed by reversed-phase High Performance Liquid Chromatography (HPLC) system (Waters, USA) on a Nucleosil C18 column (Macherey Nagel, Germany). The mobile phase was a mixture of acetonitrile and 0.05 M phosphate buffer (pH= 3; 35:65 v/v) with the flow rate of 0.7 mL/min. Detection was carried out at 287 nm with 20-50 *μ*L injection volume.

The in-vitro dissolution studies of selected formulations and two physical mixtures (PMs) were performed by dissolving the same amount of samples (700 mg) in pure water previously heated to 37 °C. 100 *μ*L of samples were collected in 0, 5, 10, 25, 35, 60, and 90 min. Afterwards, samples were analyzed by mentioned HPLC method.

## Results and Discussion

**Figure 2:**
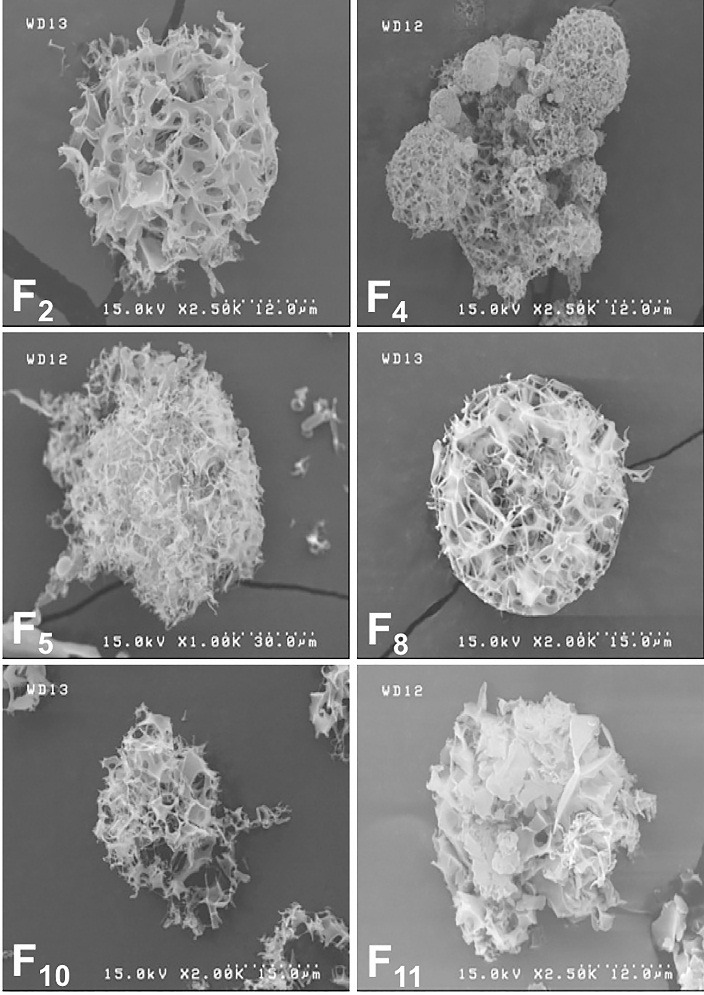
SEM images of Raloxifene Hydrochloride-loaded samples after spray freeze drying. The porous structure of the microparticles is shown in these SEM images.

Series of microparticles containing solid dispersions of RH, HPβCD or PVP were produced by SFD process according to Table 1. Micrographs of the resultant particles (Figure 2) showed numerous pores in the structure of spherical microparticles that could be attributed to the voids remained after sublimation of ice crystals. This process showed that *via* SFD technique, the atomized droplets were completely frozen in contact with liquid nitrogen and their shapes as well as sizes were preserved during freeze drying. Morphology and particle size of the microparticles were in a similar range in samples containing PVP and HPβCD. In terms of particle size, comparisons between F_2_ and F_5_ revealed that increasing the solution flow rate may result in formation of asymmetrical particles. The same results were obtained when F_8_ and F_11_ were compared. Higher flow rate may affect degree of atomization and level of freezing in droplets.

**Figure 3:**
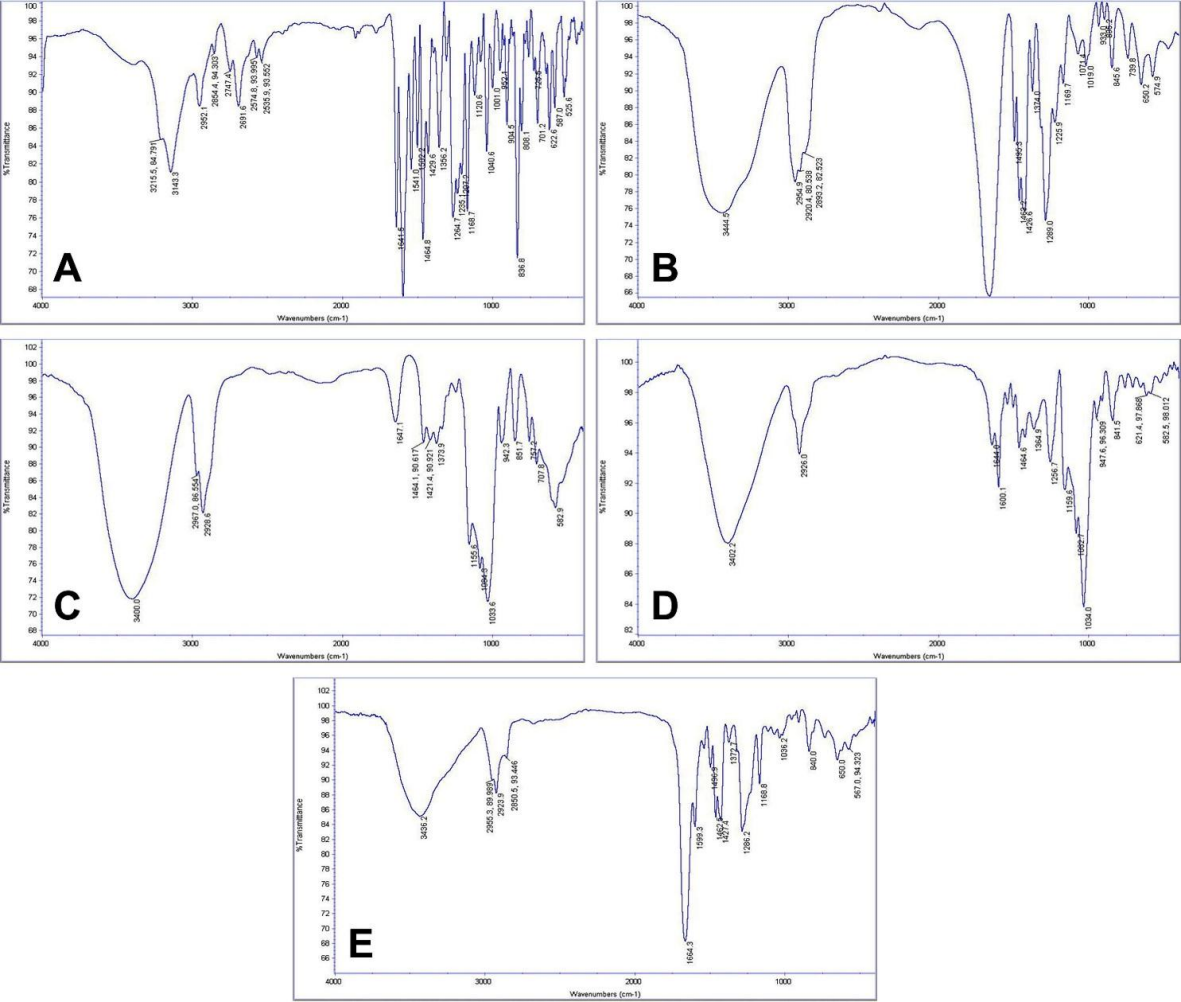
FTIR spectra for different formulations. (A) Raloxifene Hydrochloride, (B) PVP, (C) HPβCD, (D) F_4_, and (E) F_11_.

FTIR studies indicated that there was no important interaction between RH and solubility enhancers. As shown in Figure 3, the amide bond of PVP at 1690 cm^-1^ overlapped with carbonyl bond of RH; however, the stretch of C-O-C bond of RH was observed in 1600 cm^-1^ of F_11_ spectra. The broad 3400 cm^-1^ stretch and 1034 vibrating wave in F_6_ spectra confirmed the presence of hydroxyl group in HPβCD. Similarly, the 1462 cm^-1^ stretch in S-benzothiofuran of RH was observable in F_5_ sample. No wave shift was seen with pure HPβCD spectrum.

**Figure 4:**
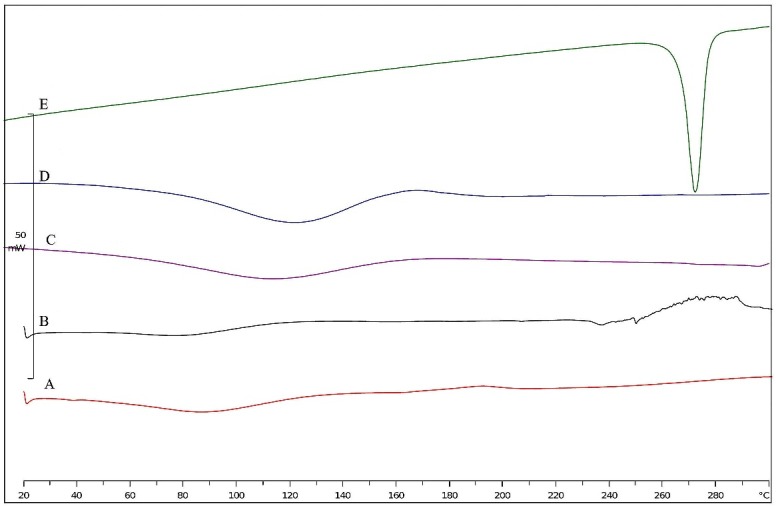
DSC thermograms for different formulations. (A) F_2_, (B) F_8_, (C) HPΒCD, (D) PVP, and (E) Raw Raloxifene Hydrochloride.

Thermograms of F_2_ and F_8_ formulations were compared with unprocessed RH, PVP, and HPβCD to investigate the thermal behavior of SFD processed microparticles (Figure 4). Pure RH exhibited a single sharp endotherm at 267 °C that was completely disappeared in both SFD processed samples and revealed amorphous state of the drug in SFD processed particles. Rapid freezing and high sublimation rate in drying step caused formation of amorphous solid dispersion in SFD method.^26^

**Figure 5:**
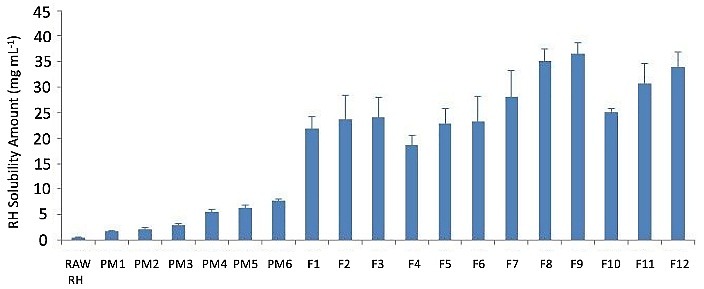
Water solubility of spray freeze dried formulations compared with Raw Raloxifene Hydrochloride and physical mixtures (PMs). PM_1_ (Raloxifene Hydrochloride:HPβCD 1:2), PM_2_ (Raloxifene Hydrochloride: HPβCD 1:3), PM_3_ (Raloxifene Hydrochloride: HPβCD 1:4), PM_4_ (Raloxifene Hydrochloride:PVP 1:2), PM_5_ (Raloxifene Hydrochloride:PVP 1:3), and PM_6_ (Raloxifene Hydrochloride:PVP 1:4). The highest water solubility of the samples was observed in F_9_ formulation.

The solubility saturation of the processed samples was compared with raw RH and physical mixtures in Figure 5. In all SFD processed samples, the solubility of drug was extremely higher than pure RH and their physical mixtures. Amorphous and porous nature of the particles can improve wettability and solubility of insoluble drugs.^15^ Higher ratios of solubility enhancers caused higher solubility levels; however, samples containing PVP showed considerably higher solubility versus HPβCD containing microparticles. The highest solubility of the samples was observed in F_9_ formulation with RH concentration of 36.65 mg/mL in comparison to 24.15 mg/mL in F_3_. This difference could be attributed to the formation of intermolecular bonding between RH and PVP.^27^

**Figure 6:**
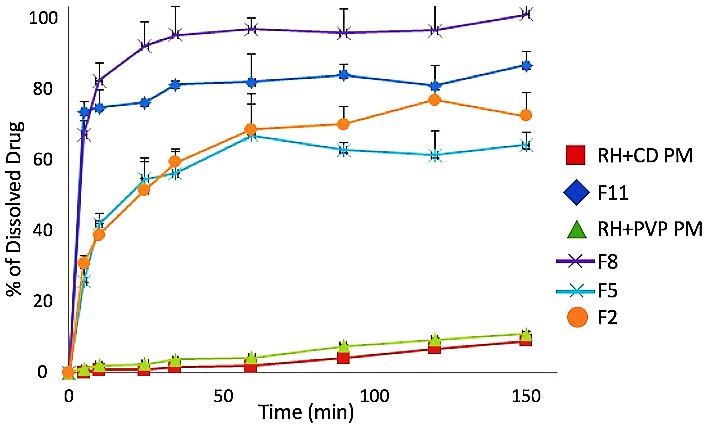
Dissolution profile of various Raloxifene Hydrochloride complexes. F_8_ and F_11_ demonstrated significantly higher dissolution rate than all the other samples and more than 83% and 77% of the drug was dissolved, respectively.

Dissolution rate of F_2_, F_5_, F_8_, F_11_, and two physical mixtures are illustrated in Figure 6. Each sample contained RH to solubility enhancer in 1:3 weight ratios. After adding the sample to the dissolution medium for 10 minutes, only 2% of RH-PVP and about 1% of RH-HPβCD physical mixtures were dissolved. On the other hand, at least 40% of the drug in SFD processed samples was dissolved within 10 minutes. Among all the samples, F_8_ and F_11_ demonstrated considerably higher dissolution rate than all the other samples and more than 83% and 77% of the drug was dissolved, respectively. These findings emphasized that dissolution rate of samples containing PVP was much faster than HPβCD processed ones due to superior wettability of PVP.

Spray freeze drying of RH with PVP and HPβCD as solubility enhancers produced amorphous and highly porous microparticles. They exhibited superior solubility and dissolution rate compared to raw RH and physical mixtures. The highest solubility was achieved by F_9_ sample with more than 70 folds increase in drug solubility. Samples containing PVP showed higher solubility versus HPβCD containing microparticles. Samples produced with lower flow rate conditions, showed higher solubility and dissolution rate.

## Conclusion

The results indicated that SFD could be an effective particle engineering approach for improving dissolution rate of poorly water soluble drugs used in oral drug delivery such as Raloxifene.

## Acknowledgments

This research was part of a PharmD student thesis (Thesis Number: 5212) and has been financially supported by Tehran University of Medical Sciences (TUMS).

## Ethical Issues

Not applicable.
